# Metabolic Syndrome: An Occupational Perspective

**DOI:** 10.4103/0970-0218.62569

**Published:** 2010-01

**Authors:** Chitra V Nair

**Affiliations:** Heavy Water Plant Kota, PO Anushakti, Via Kota, Rajasthan - 323 303, India

**Keywords:** IDF criteria, metabolic syndrome, occupational status

## Abstract

**Objective:**

To study the prevalence of Metabolic Syndrome (MS) in an Indian industrial setup and to study disparity in occurrence of MS in a working population based on occupational status.

**Materials and Methods:**

Cross-sectional study of 651 employees who underwent periodic medical examination. The International Diabetes Federation (IDF) definition of MS and International Standard Classification of Occupations (ISCO)-88 classification of occupations were used.

**Results:**

The overall crude prevalence of MS was found to be 18.5%. Nineteen percent of the non-manual workers and 18.3% of the manual workers suffered from MS. The single largest occupational category with MS was ISCO-88 group 1, which included the managers and senior officials. However, no difference was found among the manual and non-manual workers in prevalence of MS.

## Introduction

Industrialization and urbanization are contributing to a global epidemic of cardiovascular disease (CVD). Environmental factors and changes in lifestyle are major contributors. Consumption of high-calorie, refined food in association with decreased levels of physical activity is the main culprits in this global epidemic, which is affecting the developing world as well as the more prosperous developed countries.

Syndrome X (insulin resistance syndrome) or Metabolic Syndrome (MS) first described by Raeven(1) in 1988 consisted of certain factors such as hypertension, hyperlipidemia and hyperglycemia. These clustered together and were found to increase the risk of development of CVD. It was the National Cholesterol Education Program Adult Treatment Panel III (ATP III),(2) which first used the term MS as beyond CVD and type 2 diabetes. Individuals with MS seemingly are susceptible to other conditions, notably polycystic ovary syndrome, fatty liver, cholesterol gallstones, asthma, sleep disturbances and some forms of cancer. In 2005, the International Diabetes Federation (IDF) further modified the ATP III definition and suggested that criteria used for definition should be made specific in different ethnic groups. Since visceral obesity correlated well with insulin resistance, it was made an essential criterion for diagnosis [Table 1]. These criteria have been adopted recently by the Indian Council of Medical Research.

**Table 1:** International Diabetes Federation criteria for clinical identification of the metabolic syndrome in males

Risk factor	Defining level
Abdominal obesity (waist circumference)	>90 cm
Triglycerides	≥150mg/dL
HDL cholesterol	<40 mg/dL
Blood pressure or treatment of previously diagnosed hypertension	≥130/≥85mm Hg
Fasting glucose or treatment of previously diagnosed diabetes	≥100mg/dL ‡

*When criteria 1 and any two of the remaining four criteria are present, a diagnosis of metabolic syndrome can be made.

The age-adjusted risk in the Framingham offspring study with MS was 2.54 for coronary heart disease and 6.92 for diabetes in men, whereas in women, coronary heart disease risk was lower at 1.5, while diabetes risk was similar at 6.5.(3) The MS alone predicted ≈25% of all new-onset CVD. Ten-year risk in men with MS generally ranged from 10 to 20%. Prospective studies have shown that components of MS are common and are associated with incident CVD and diabetes after five years and that interventions to alter BMI, lipid levels and blood pressure may decrease incident diabetes and CVD.(4)

Early identification, treatment and prevention of the MS present a major challenge for healthcare professionals facing an epidemic of overweight and sedentary lifestyle. As the syndrome has a multifactorial and often modifiable causation, it continues to have an important role in both individual and public healthcare.(5) The chief contributing factors for development of MS were found to be advanced age, tobacco consumption, sedentary job and lack of physical exercise.

Much research has been focused on modifiable factors that may influence CVDs. Socioeconomic status may provide a new focus.(6) There exists a relationship between a lower socioeconomic status and an increased mortality and morbidity rate in the general population. Unhealthy lifestyle factors including alcohol and tobacco consumption,(7) inadequate access to quality healthcare, material deprivation and a stressful psychosocial environment have been cited to be the main contributing factors.(8) Disease-specific mortality rates indicated that members of the manual class run a higher risk of dying from ischemic heart disease, cancer and gastrointestinal diseases than those of the non-manual class.(9) Occupation may also exert a positive influence on health and survival with better income and prestige received from an occupation reflecting in better living conditions, access to quality healthcare and adoption of a healthier lifestyle.(10) However, studies have also revealed that MS is often associated with sedentary occupations.(11)

Studies have shown that there is a significant prevalence of diabetes and MS in the urban Indian population with the prevalence being age related and more so among the sedentary workers.(12–14) To date only a handful of studies have been conducted in order to assess the impact of one's occupation in development of MS.

The aim of the study was to assess the prevalence of MS in an urban Indian industrial setup and to assess the impact of occupational status in predicting the development of MS.

## Materials and Methods

This study included 651 male employees, who underwent their annual medical examination at the plant site occupational health center from June 2008 to December 2008. The medical examination included a complete physical examination and laboratory tests. Male employees in the age group of 30 to 60 years were included in the study. As we were studying the existence of risk factors in a seemingly normal population, all patients with documented CVD were excluded from the study. The study excluded subjects fulfilling the ICD 9 criteria of Ischemic Heart Disease (codes 410-414) and cerebrovascular diseases (codes 430-438). Women could not be included as the number of women was far too small (*N*<10) to form a representative sample.

All anthropometric measurements were made after 12 h of fasting, with the participant wearing light clothing. The abdominal circumference was measured midway between the inferior margin of last rib and iliac crest in the mid-axillary plane at the end of normal relaxed exhalation with a flexible and non-distendable tape. Blood pressure was measured with a standard mercury sphygmomanometer. Two blood pressure readings were taken with the participant resting for 10 min, and the mean of the two readings calculated. Blood was sampled after a 12-h overnight fast. Glucose, high density lipoprotein (HDL) and triglycerides were measured using automatic standard enzymatic methods.

The IDF definition of MS was used.^#^ Using the International Standard Classification of Occupations (ISCO)-88,(15) major groups 1 to 4 were classified as non-manual workers, who had higher levels of education and better occupational profiles including managers, scientists, accountants administration and paramedical personnel, while major groups 5 to 9 were classified as manual workers with lower levels of education and lower income levels and included drivers, assistants, helpers, tradesmen, firemen, canteen, housekeeping and general service workers. International Standard Classification of Occupations group 6 of agricultural and fishery workers are not employed in this particular industry.

## Results

The overall prevalence of MS was 18.5%. The average age of the employees with MS was 49.43 (95% Confidence Interval 50.22–48.64). The prevalence of MS is 2.2% among individuals aged 30–40 years, 15.8% among the 41–50 year age group and 29.51% among individuals aged 51–60 years. Hence it confirms with the worldwide trend of increasing prevalence of MS with an increase in age.

The average blood pressure of the workers with MS was systolic 131.33 (95% Confidence Interval 135.73–130.93) and diastolic 84.90 (95% Confidence Interval 86.2–83.6). The average fasting blood glucose was 107.63 (95% Confidence Interval 114.37–100.89). The average abdominal circumference was 97.65 (95% Confidence Interval 100.35–94.95). Among the men with central obesity, 80.1% had hypertension or were taking anti-hypertensives. Approximately 66.9% had elevated fasting blood glucose or were taking diabetes medications. About 45.1% had elevated triglycerides and 38% had low HDL levels as per the IDF criteria. It was found that 31.1% of the subjects with MS reported using tobacco on a regular basis.

Of the 651 employees examined, 19% of the non-manual workers and 18.3% of the manual workers suffered from MS. The single largest occupational category with MS was ISCO-88 category 1, which are the managers. The difference in prevalence of MS among the manual and non-manual workers was statistically insignificant on application of Chi-square test [Table 2].

**Table 2:** ISCO-88 occupational category and prevalence of metabolic syndrome among male workers aged 30-60 years

Job profile	ISCO occupational group	Prevalence of metabolic syndrome
Managers and senior officials	1	30.7
Scientific professionals (engineers, physicists, chemists)	2	18.4
Associate professionals (Paramedical professional, finance and associate professionals)	3	11.11
Clerks	4	24.0
Service workers (Housekeeping, canteen workers)	5	12.0
Trade-related workers (Machinery and electrical equipment, mechanics, fitters)	7	18.75
Plant and machine operators, motor vehicle drivers	8	21.17
Elementary occupations (helpers, messengers, cleaners)	9	15.7

## Conclusion

The study showed that the prevalence of MS increased strongly with age. In this study group, among all the variable components of MS, hypertension was found to be most prevalent followed by elevated fasting blood glucose.

The prevalence of MS varied in the different categories of occupational activity. The single largest occupational category with MS was ISCO-88 category 1, which are the managers. Neither education nor occupational status was significantly associated with the prevalence of MS among our male participants.

Since many of these factors are modifiable, intensive efforts targeted towards the population at risk including health education programs, periodic health check-ups, prompt treatment and referral of cases are called for. The AHA/NHL1 scientific statement has recommended that ‘to reduce the lifetime risk of atherosclerotic CVD, all individuals found to have MS deserve long-term management and follow-up in the clinical setting’. As many an employee spends a lifetime in an organization, an industrial setup provides an excellent avenue for diagnosis, primary prevention and long-term management of subjects with MS.
